# Structured-Light-Based System for Shape Measurement of the Human Body in Motion

**DOI:** 10.3390/s18092827

**Published:** 2018-08-27

**Authors:** Paweł Liberadzki, Marcin Adamczyk, Marcin Witkowski, Robert Sitnik

**Affiliations:** Institute of Micromechanics and Photonics, Faculty of Mechatronics, Warsaw University of Technology, ul. Św. Andrzeja Boboli 8, 02-525 Warsaw, Poland; m.adamczyk@mchtr.pw.edu.pl (M.A.); m.witkowski@mchtr.pw.edu.pl (M.W.); r.sitnik@mchtr.pw.edu.pl (R.S.)

**Keywords:** 4D imaging, whole-body scanning, structured light, single frame

## Abstract

The existing methods for measuring the shape of the human body in motion are limited in their practical application owing to immaturity, complexity, and/or high price. Therefore, we propose a method based on structured light supported by multispectral separation to achieve multidirectional and parallel acquisition. Single-frame fringe projection is employed in this method for detailed geometry reconstruction. An extended phase unwrapping method adapted for measurement of the human body is also proposed. This method utilizes local fringe parameter information to identify the optimal unwrapping path for reconstruction. Subsequently, we present a prototype 4DBODY system with a working volume of 2.0 × 1.5 × 1.5 m^3^, a measurement uncertainty less than 0.5 mm and an average spatial resolution of 1.0 mm for three-dimensional (3D) points. The system consists of eight directional 3D scanners functioning synchronously with an acquisition frequency of 120 Hz. The efficacy of the proposed system is demonstrated by presenting the measurement results obtained for known geometrical objects moving at various speeds as well actual human movements.

## 1. Introduction

Over the last few years, substantial improvements in three-dimensional (3D) scanning technology have increased the popularity of this method for fast and accurate surface reconstruction. Three-dimensional scanning technology is employed in many science and technology fields for a variety of purposes, including law enforcement work, cultural heritage investigations, medicine and entertainment. In law enforcement, 3D scanners are used for crime scene reconstruction [1,2] and enable further investigation, such as bloodstain pattern analysis [3], to be conducted. In cultural heritage studies, this technology can be used to characterize cultural heritage objects completely, including their shapes and colours [4,5]. In some cases, the reconstruction is performed with the use of additional information from multispectral analysis [6,7]. In the medical field, 3D scanning technology is employed for many different purposes [8], including body surface analysis for anatomical structure detection [9,10] and internal body analysis [11]. Three-dimensional scanners are also utilized in entertainment to capture accurate and realistic 3D textured models for computer graphics [12,13].

In this paper, we focus on the acquisition of comprehensive measurements of a full human body. Currently, the most popular full-field measurement techniques used for 3D human surface acquisition are the laser triangulation (LT) [14,15], time of flight (TOF) [16,17], structured light (SL) [18,19] and structure from motion (SfM) [20,21] methods. Most of the available scanning techniques are only intended to measure the human figure in static poses.

When considering 3D scanning of the human body, integrating the fourth dimension, time, presents an opportunity for improvement. Data captured over an extended period of time could easily be incorporated to enhance practical applications such as medical diagnostics and computer graphics. The lack of four-dimensional (4D) information, that is, 3D data obtained over an extended period of time, limits the applications of many existing 3D scanning techniques. Although these techniques are adequate for 3D data and each technique can be used to obtain dynamic measurements in specific conditions, the existing approaches all encounter difficulties when capturing 4D information. For example, motion capture (mocap) systems are commonly used to gather similar types of data (i.e., 4D data) but instead of the whole surface geometry, mocap systems only track the positions of certain points [22,23].

Next, we review the existing full-field measurement methods considering the objective of delivering surface information that extends beyond fiducial landmarks. Table 1 presents the advantages and disadvantages of the existing full-field techniques in 4D measurement applications. The SL technique is predominantly based on the projection of a sequence of certain pattern images. Thus, the temporal consistency of an object within a single measurement is required to obtain accurate results. Several reports have suggested that the optimal 4D measurement approach should be based on the projection of a single frame pattern [24,25]. This variant of the SL technique encounters two problems. First, when phase reconstruction relies on data from a single frame, the resulting high error rate diminishes the quality of the generated models. Second, phase unwrapping is impossible without additional information, which creates the problem of appropriate modification of the projected pattern [26,27].

Alternatively, SfM algorithms consider multiple images of the different sides of the measured object. Real-time scanners based on this technique must include numerous devices to provide complete sets of synchronously captured images. This approach is encumbered by extremely time-consuming data post-processing, and, depending on the density of unique surface features in the images, the local surface reconstruction quality may fluctuate [28,29].

TOF cameras are commonly used to gather dynamic (4D) data rather than static data. Although TOF cameras are fast, the captured human body surface measurements are often inaccurate [30,31].

LT can be applied to dynamic shape measurements under the condition that multiple laser stripes are used. This approach is problematic because the resulting body surface sampling rate varies with the projected pattern direction [32,33]. For example, if the lines are projected horizontally, then spatial resolution in the vertical direction is much lower than that in the horizontal direction.

In this paper, we present a 4D measurement system (called 4DBODY) that was developed for imaging the human body surface while in motion. The SL method is employed in the proposed system, because this approach provides high-resolution 3D geometry measurements together with relatively high measurement speed and accuracy. We defined three goals that should be achieved to enable the use of measurement data in practical applications. The first goal was full-body measurement, with minimal uncovered areas. The second one was a sampling frequency around 100 Hz when capturing the human body in motion. This value is acceptable for most human movements. We adjusted the sampling frequency up to 120 Hz in this study due to the technical parameters of the available digital projectors. The third goal was the definition of the output data as a cloud of points in terms of spatial resolution and measurement uncertainty. The spatial resolution was defined as the maximum distance between neighbouring points and the measurement uncertainty was defined as the acceptable error of the location of each point (*x*, *y*, *z*). We assumed that the spatial resolution and measurement uncertainty should be equal to or less than 1.0 mm and 0.5 mm, respectively. These values enable rendering of the resultant data without interpolation in standard, full high definition (HD).

The remainder of this paper is organized as follows. Section 2 summarizes the previous studies and outlines their main points and limitations. Section 3 introduces the system concept as well as the system structure and its components and Section 4 describes the developed method and explains the system calibration process. To confirm the reliability of the proposed system, Section 5 discusses the system validation and shows exemplary results. Finally, Section 6 summarizes the study.

## 2. Previous Works

The methods mentioned in Section 1 are used in some existing systems to obtain dynamic shape measurements. For instance, Lenar et al. [27] used a measurement system based on SL called OGX|4DSCANNER for real-time evaluation of lower body kinematics. A single frame pattern and a fringe projection method are employed in this system and the absolute phase distribution is reconstructed based on a single fringe with a known absolute phase value. Two-directional measurements of the lower human body can be obtained using four detectors and two pattern projectors. This system utilizes only two measurement directions, with no overlap of the projected patterns. In their research, Lenar et al. successfully demonstrated the suitability of 4D surface data for musculoskeletal analysis. However, the utilization of only two measurement directions leads to large areas with no data, which is unacceptable for most applications. Imaging only the lower part of the body is a significant limitation as well.

Using 3dMD technology for research on soft-tissue deformations, Pons-Moll et al. [34] created a model for predicting these deformations. Zhang et al. [35] then used that system in their research on estimating body shapes under clothing. The system utilized by Zhang et al. consists of 22 pairs of stereo cameras, 22 colour cameras, 34 speckle projectors and arrays of white-light light-emitting diode (LED) panels. It can capture 3D full-body scans at 60 Hz but the projectors and LEDs flash at 120 Hz to alternate between stereo capture and colour capture. However, the effectiveness of the 3dMD system comes with the trade-off of expensiveness. 

In another approach, a Microsoft Kinect v2.0 depth sensor was employed [36]. The acquired 4D data were used for augmented reality to visualize the fit of clothing to the human body in images that followed the movements of customers. As this system is single-directional, this approach cannot be used to capture complete 360° scans. Moreover, the spatial accuracy of the Kinect sensor and its acquisition rate of 30 Hz [37] may be insufficient for dynamic human body measurements.

The biomedical technology manufacturer DIERS International GmbH proposed a system [38] for dynamic spine and posture measurements, which consists of a single detector–projector pair and primarily relies upon SL and LT to perform back reconstruction, in which the position of the spine of the patient is adjusted according to the surface topography generated based on a mathematical model. Gipsman et al. [32] and Betsch et al. [33] used DIERS International GmbH technology in their research to verify the applicability of the system to spine curvature analysis. However, complete 360° scans cannot be captured using this system.

Brahme et al. [39] presented a system for 4D patient pose estimation during diagnostic and therapeutic procedures. This system is based on LT and involves the use of multiple laser stripes with a measured volume of approximately 40 × 40 × 20 cm^3^. Although multiple laser stripes can be employed to capture simultaneous scans of the entire measurement area, the spatial resolutions of these data are uneven because, while high resolution can be achieved in the direction of the laser stripes, the areas between these stripes exhibit low resolutions.

Collet et al. [40] proposed an integrated approach that combines three different techniques for 4D reconstruction: shape from silhouette, SfM and infrared SL. The final model is represented by an animated 3D mesh textured by its natural red, green and blue (RGB) colour. This solution is complete and mature from technical and practical perspectives but it is very expensive and requires the use of a green-box.

These systems have numerous limitations such as small measurement volume, limited number of measurement directions, low acquisition frequency, or high cost. In this paper, we address these limitations with an approach extended to 4D body scanning, which is partially based on the method previously proposed by Lenar et al. [27].

## 3. Acquisition System Design

**Figure 1:**
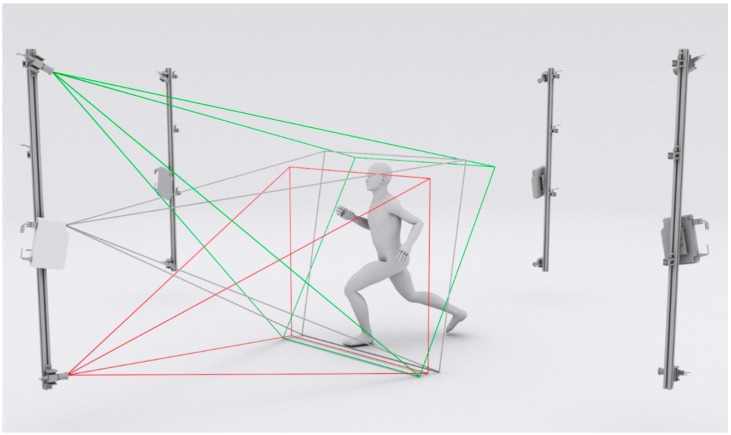
Architecture of the proposed acquisition system.

We decided to use four directional measurement heads (DMHs) evenly distributed around the measurement volume, as a compromise between the completeness of the final model and the equipment cost. This setup provides almost complete reconstruction, excluding some areas around the armpits and groin. Increasing the number of DMHs would not significantly affect the areas in these regions that are not measured but would increase the equipment cost. The resultant measurement volume is 2.0 × 1.5 × 1.5 m^3^. To reconstruct as much of the human body surface area properly as possible without skipping portions such as the shoulders or perineum region, two detectors and one projector are used in each DMH, as illustrated in Figure 1.

**Figure 2:**
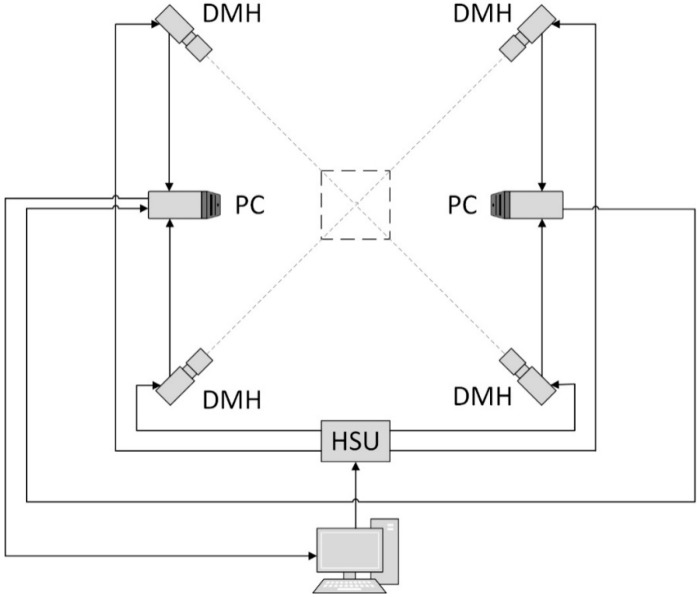
Communication model used in the proposed system.

**Figure 3:**
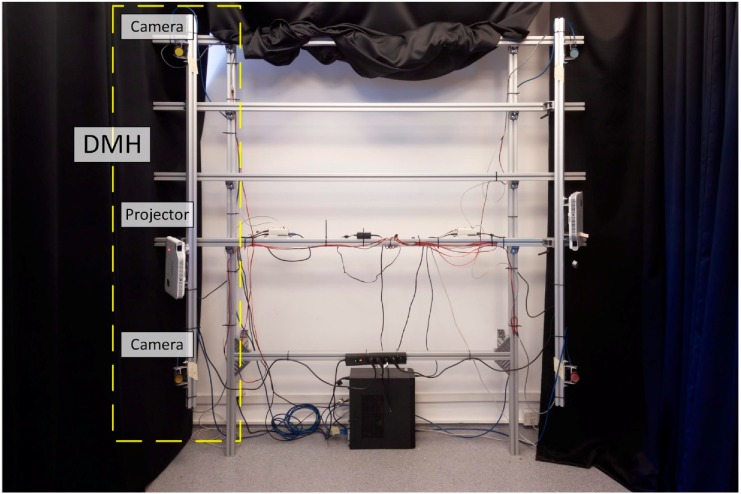
Photograph of the developed 4DBODY system (two directional measurement heads (DMHs) are visible). A single DMH with its constituent devices is enclosed within the dashed rectangle.

The detectors are Grasshopper 3.0 cameras with 2.3 megapixel Sony sensors, 163 Hz capture frequencies in free-run mode and 120 Hz capture frequencies in synchronous mode [41]. Each DMH includes one Casio XJ-A242 projector (1280 × 800 pixels, 2500 ANSI LUMENS) with two light sources, an LED for the R channel and a laser for the B and G channels [42]. To avoid crosstalk between the cameras and projectors of the neighbouring DMHs, spectral separation is applied. Each DMH uses one R, G, or B channel for projection. The corresponding spectral filters are mounted on the camera lenses. Figure 2 depicts the two personal computers (PCs) and the single custom-designed hardware synchronization unit (HSU) that controls the proposed system. Each PC is responsible for the management and acquisition of two DMHs. The HSU is responsible for the synchronous projection-acquisition of all of the DMHs. This task is realized by a wired synchronization connection between the projector, cameras and HSU. A photograph of one side of the developed measurement system is presented in Figure 3. Aside from the projectors, the room with the system does not contain any other light sources. We used additional blackout curtains to provide the highest possible fringes modulation and remove influence of external lighting. The curtains are outside of the measurement volume; thus, they do not appear as part of the measurements.

To summarize, the 4DBODY system was designed to realize synchronous projection and acquisition of images with a frequency of 120 Hz, which is twice as high as the frequencies of the systems presented in Section 2. Compared to the other systems that can provide full human body measurements, this system can capture a similar amount of data. However, due to the use of only four measurement directions, the cost of the equipment in our system is significantly lower that it is for the other described systems. A comparison of the proposed acquisition system with the previous systems is presented in Table 2.

## 4. Measurement Process

The entire 4DBODY system must be calibrated before measurement. The same single-frame processing method is used in the calibration and measurement procedures. In the following subsections, we will describe the single-frame processing method followed by the calibration of the whole system.

### 4.1. Single-Frame Processing

**Figure 4:**
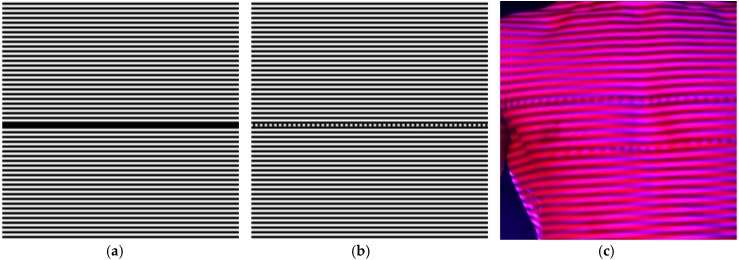
Projected patterns: (**a**) original Sitnik pattern from [43]; (**b**) modified pattern presented in this paper; and (**c**) two spectrally separated patterns (red and blue) projected onto a human body surface.

To avoid motion artefacts, a single frame method is used in the 4DBODY system for shape measurement. For this purpose, we modified the single sine pattern method proposed by Sitnik [43]. The modification involves using a different design for a single distinguished fringe, which is modulated transversely for fringe orientation in the proposed technique, as displayed in Figure 4. This distinguished fringe is called the marker and is used to determine the absolute phase value. During image analysis, the marker is localized in the image space by performing one-dimensional (1D) fast Fourier transform (FFT) frequency filtering [44]. The spatial-carrier phase-shifting (SCPS) method proposed by Larkin [45] is employed to calculate a modulo-2π phase. The seven-point method was selected as a compromise between phase quality and minimal analysed fringe period. Furthermore, the selected SCPS method is resistant to inaccurate intensity sampling within the area of a single fringe period.

**Figure 5:**
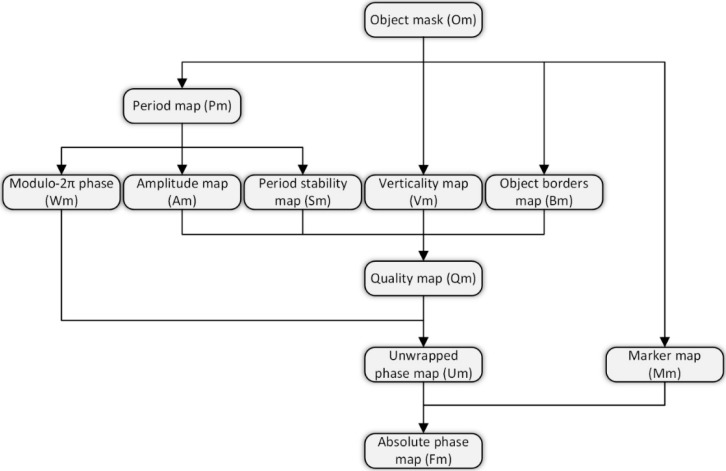
Single-frame processing scheme.

One of the most difficult aspects of the SCPS method is determining a phase unwrapping technique that is reliable, especially in areas in which the local shape derivative is discontinuous. When applying phase unwrapping to the human body, problems usually arise in several regions, specifically, the cervical (neck), mammary (breast), axillary (armpit), antecubital (elbow), inguinal (groin) and popliteal (knee pit) regions. Thus, the proposed method is focused on identifying areas to perform phase unwrapping reliably and accurately. This objective is realized by creating a quality map (Qm) enabling proper phase unwrapping, based on a reliable spanning tree [46]. The general processing path of a single frame is presented in Figure 5. A brief explanation of Figure 5 is given next, followed by a more detailed description of the algorithms used in the proposed approach.

The first processing step involves separating the analysed surface from the background using an object mask (Om). The Om limits the subsequent calculations to only the object area, eliminating erroneous off-object areas and dramatically increasing the processing speed. In the next step, the fringe period per pixel map (Pm) and modulo-2π phase map (Wm) are calculated. To achieve the final Qm values, the following two-dimensional maps are calculated:

- Fringe amplitude map (Am)—favours areas with high fringe contrast, eliminating errors due to incorrect fringe period estimation;
- Period stability map (Sm)—favours areas with stable fringe periods, avoiding areas with local period discontinuities;
- Fringe verticality map (Vm)—favours areas consisting of fringes with locally constant orientations, according to the projected orientation, thus avoiding high curvature areas;
- Border areas map (Bm)—favours areas with the greatest distances to the edges of the object, thus eliminating errors due to surface discontinuities.

The Qm calculation method is heuristic and is adjusted to achieve proper human body measurements. The calculated spanning tree is applied to the Wm, with branch weights based on the Qm, to derive the Um. Then, the Um and Mm, which provide information about the marker location, are used to generate the final absolute phase map (Fm).

**Figure 6:**
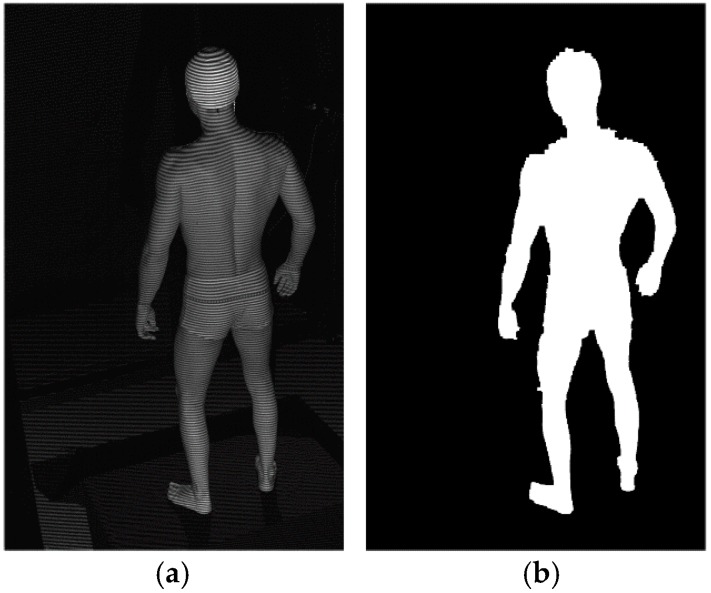
Example images depicting the Om calculation procedure: (**a**) input image; (**b**) output image.

As shown in Figure 6, the Om calculation begins with the masking of overexposed pixels and Otsu thresholding [47]. Next, dilation and erosion are applied to produce smooth contours. Finally, the largest segment in the image is selected and everything else is masked.

**Figure 7:**
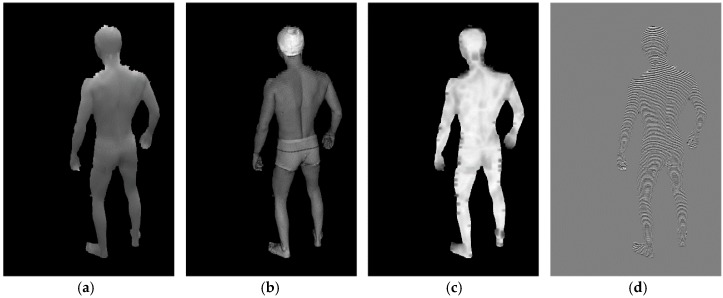
Example images depicting certain maps calculated in this study: (**a**) Pm; (**b**) Am; (**c**) Sm; (**d**) Wm.

The corresponding Pm and Am are calculated for each object pixel in the Om and are derived from the median, maximum and minimum values of the local intensity in the neighbourhood. To calculate the Pm, such as that shown in Figure 7a, the median intensity is used for the thresholding of the local intensities as well as for counting the period values in the directions perpendicular to the fringes. The Am values in Figure 7b represent the differences between the local maximum and minimum intensities. Next, the Sm, such as that presented in Figure 7c, is calculated as the variance of the Pm in the direction of estimation. The Wm, such as that depicted in Figure 7d, is calculated for each object pixel in the Om using the Pm values to select samples based on the local fringe period. Linear interpolation is used for non-integer coordinate sampling.

**Figure 8:**
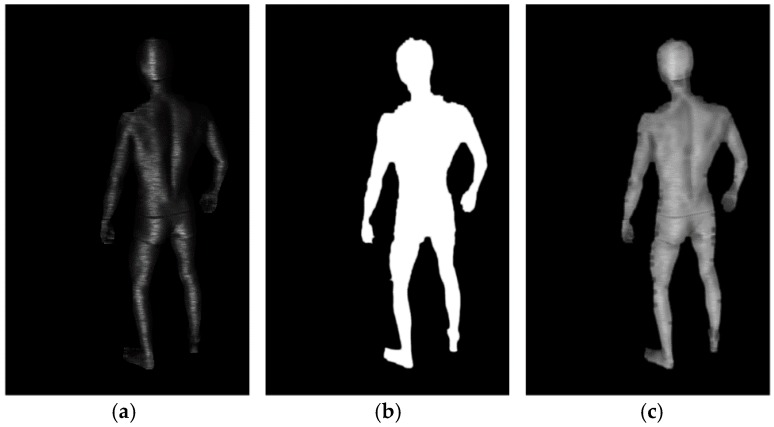
Example images showing the (**a**) Vm and (**b**) Bm calculation results and (**c**) Qm calculated based on the Vm and Bm, together with the Am and Sm.

Next, the Vm, such as that in Figure 8a, is derived based on the calculated intensity gradients in both the vertical and horizontal directions. The quotient of the horizontal gradient over the vertical gradient can be taken as a measure of fringe verticality, as detailed in Equations (1)–(3). The window size in pixel (*r*, *c*) is equal to local fringe period taken from the Pm.

(1) $$grad_{V}\left(r,c\right)=\sum_{i=r+1}^{r+w}\left|I\left(i,c\right)-I\left(i-1,c\right)\right|+\sum_{i=r-w}^{r-1}\left|I\left(i,c\right)-I\left(i+1,c\right)\right|$$

(2) $$grad_{H}\left(r,c\right)=\sum_{j=c+1}^{c+w}\left|I\left(r,j\right)-I\left(r,j-1\right)\right|+\sum_{j=c-w}^{c-1}\left|I\left(r,j\right)-I\left(r,j+1\right)\right|$$

(3) $$grad_{H}\left(r,c\right)=\sum_{j=c+1}^{c+w}\left|I\left(r,j\right)-I\left(r,j-1\right)\right|+\sum_{j=c-w}^{c-1}\left|I\left(r,j\right)-I\left(r,j+1\right)\right|$$

where

r and c: row and column number, respectively, of the central pixel;
i and j: row and column number, respectively, of the current pixel;
w: window size;
I(r,c): intensity of pixel (r,c) in the image;
$grad_{V}\left(r,c\right)\;\mathrm{and}\;grad_{H}\left(r,c\right)$: vertical and horizontal gradients, respectively, of pixel (r,c);
Vm(r,c): verticality of pixel (r,c) in the Vm.

As shown in Figure 8b, the Bm calculation simply blurs the Om. The Bm is used to reduce the weights of the pixels near the border between object and background, which is a highly erroneous area. Together with the Am and Sm, the Vm and Bm are normalized and used to construct the Qm, such as that depicted in Figure 8c. Equation (4) is used to evaluate the quality as the weighted arithmetic mean of the powers of the pixel values from the four contributing maps. The weights that we established experimentally, enabling us to achieve reliable results, were as follows: $b_{w}=1,\;a_{w}=5,\;v_{w}=3,$ $s_{w}=1,\;b_{e}=1,\;a_{e}=2,\;v_{e}=1,\;s_{e}=1$, where these variables are as defined after Equation (4). According to our experience, the Am map has the greatest influence on the proper unwrapping procedure. Additional power weights enable the gradients of values in a particular map to be increased, leading to less error-prone unwrapping of the human body geometry. The weight values were established in this study for human body measurement and should be adopted for other subjects.

(4) $$Qm\left(r,c\right)=\frac{b_{w}*Bm{\left(r,c\right)}^{b_{e}}+a_{w}*Am{\left(r,c\right)}^{a_{e}}+v_{w}*Vm{\left(r,c\right)}^{v_{e}}+s_{w}*Sm{\left(r,c\right)}^{s_{e}}}{b_{w}+a_{w}+v_{w}+s_{w}}$$

where

r and c: row and column, respectively, of a pixel;
$b_{w},\;a_{w},\;v_{w}\;\mathrm{and}\;s_{w}$: weights of the Bm, Am, Vm and Sm components, respectively;
$b_{e},\;a_{e},\;v_{e}\;\mathrm{and}\;s_{e}$: exponents of the Bm, Am, Vm and Sm components, respectively;
Bm(r,c), Am(r,c),Vm(r,c) and Sm(r,c): pixel values in the Bm, Am, Vm and Sm, respectively;
Qm(r,c): quality value of pixel (r,c).

**Figure 9:**
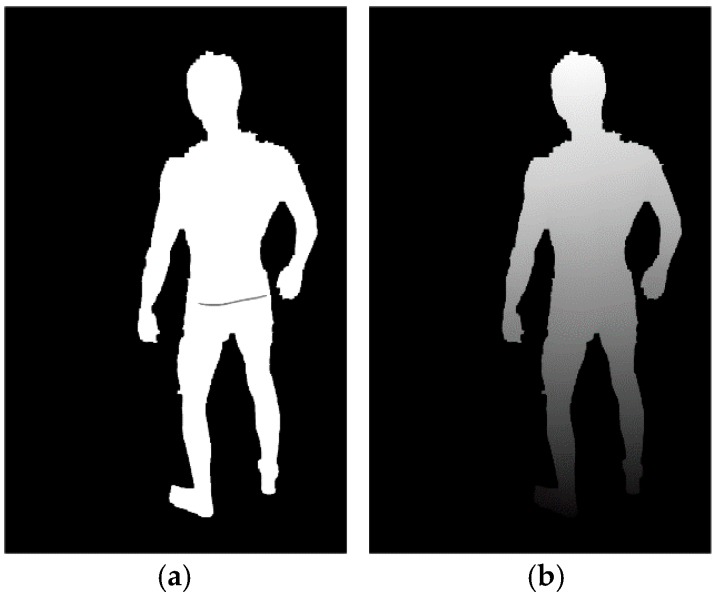
Visualization of the last steps of single-frame processing: (**a**) Mm; (**b**) Fm.

The Um calculation begins by selecting a random object pixel from the top 10% of the Qm as the initial point for the minimum spanning tree algorithm [46]. Therefore, the calculated Um must be shifted by a certain value to obtain the absolute phase distribution. The Mm, which contains information about the distinguished fringe location, is used to determine the value of the shift and thus to construct the final output, the Fm. An example Fm obtained in this study is depicted in Figure 9b. The Mm calculation begins by performing one-dimensional (1D) FFT [44] to filter out frequencies with orientations similar to those of the calculated fringes. Subsequently, after conducting an adequate inverse FFT [44], thresholding and segmentation are performed to identify the largest segment representing the marker, as shown in Figure 9a. Marker pixels are used to calculate the marker phase value, thereby providing the marker fringe number. Then, the median of the phase values under the marker pixels is obtained and the necessary phase shift is calculated by applying Equation (5):

(5) $$\Phi_{x}=2\pi *\left[N-floor\left(\frac{median+\pi }{2\pi }\right)\right],$$

where

N: projected marker index;
$\Phi_{x}$: phase shift.

Later, the Fm is scaled to a point cloud in real-world coordinates (*x*, *y*, *z*) based on phase/geometry calibration data from the DMHs. The next section describes the calibration procedure for a single DMH and the global relative calibration of all of the measurement modules.

### 4.2. Calibration Procedure

The procedure for calibrating the proposed 4DBODY system has three stages. In the first stage, both detectors of each DMH are calibrated together, with each DMH being calibrated independently. In the next stage, phase calibration is conducted for each projector–detector set that constitutes a single DMH. The camera and phase calibration of the DMHs enables every DMH to collect measurements independently. The final calibration stage, called global calibration, involves calculating transformations between individual module measurement volumes, enabling (*x*, *y*, *z*) data to be received in the common, global coordinate system.

**Figure 10:**
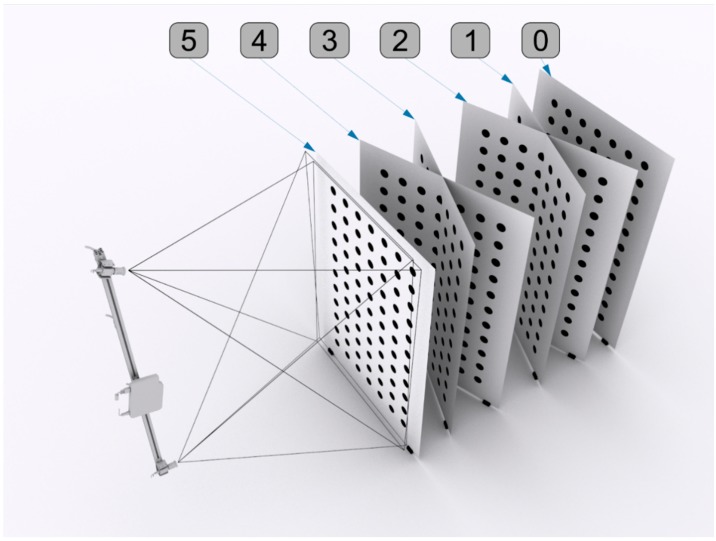
Single DMH calibration process: calibration artefact positions 0–5 used during camera calibration.

The first two calibration stages pertain to the local calibration of individual DHMs, as conveyed in Figure 10. These two stages are executed according to the procedure described by Sitnik [48]. A dedicated calibration artefact is employed, which is validated using a coordinate measuring machine (CMM) and has the form of a white board with black, circular markers aligned in rows and columns. The dimensions of this artefact are 2.0 × 1.5 m^2^. During camera calibration, a line in the 3D coordinate system is assigned to each detector pixel. Subsequently, the real x and y coordinates are determined for each pixel. The phase calibration is based on triangulation with the distinction that, in the proposed method, the projector acts as the second device in the triangulation pair. This alteration produces a phase-to-depth distribution for each detector pixel. This distribution, together with the 3D lines from camera calibration, constitutes a mapping from pixel coordinates and pixel absolute phase values to a specific 3D point in the real coordinate system. Thus, the Fm calculated from a single image is processed into a 3D reconstruction of a measured object. The output data are presented in the form of a cloud of points. In addition to the (*x*, *y*, *z*) coordinates, the output contains two more data buffers in the individual points: intensities and normal vectors. The normal vectors are calculated based on the (*x*, *y*, *z*) coordinates of the neighbouring detector pixels for each point in the cloud. This form of the output data was selected owing to its suitability for further processing and conversion into other forms, such as that used in the triangle mesh model.

**Figure 11:**
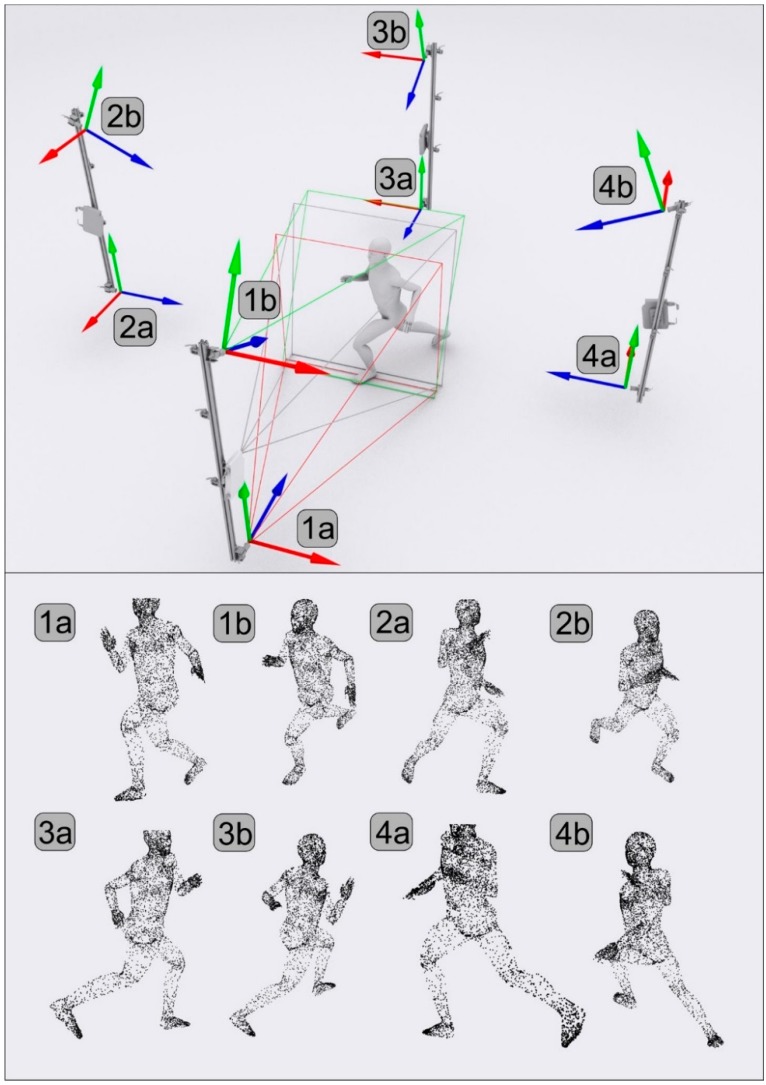
Integration of the four DMH coordinate systems using the camera coordinate systems depicted in 1a–4b and respective point clouds. Note that the number of points was reduced to improve the clarity of the figure.

Two directional point clouds generated by a single DMH are spatially aligned with each other. However, as the clouds generated by different DMHs are defined by different, local coordinate systems, global calibration is performed to realize proper, integrated multiview measurement. The same calibration artefact employed for local calibration is used for global calibration. The calibration artefact is placed in the measurement volume in a position that is visible to the detectors of two neighbouring DMHs. The calibration pattern positions expressed in the local coordinate system of each DMH are analysed and used to estimate the mutual transformations between the two devices. This process is executed on each possible pair of DMHs, thereby capturing all of the relative transformations. Thus, the four sets of individual point clouds, as exhibited in Figure 11, can be merged into a single multidirectional cloud by applying the calculated transformations only. The final measurement results are represented by all of the points from the directional clouds of points with the corresponding 3D transformations.

## 5. Validation of the Proposed System

### 5.1. Initial Validation

After completing the calibration process, the accuracy of the overall 4DBODY system was evaluated. Generally, SL scanners are validated according to the recommendations in VDI/VDE 2617-6 [49]. Considering the dynamic aspects of the measurements performed using the proposed system, we developed a simplified and adapted approach based on the measurement of an object with a known geometry. The calibration artefact was also used in this initial validation. The artefact was attached to a turntable, which allowed us to examine both static and dynamic cases using stable and rotating models, respectively. We tested model rotation speeds of 4.0 rpm, that is, approximately 0.20 m/s on the side of the model used; 9.0 rpm, or approximately 0.45 m/s; and 14.0 rpm, or approximately 0.70 m/s. For each case, validation was executed separately for each DMH. The validation process consisted of two error analysis steps.

- Step 1: A virtual plane was fit to the received cloud of points, that is, the captured model.
- Step 2: The distances between the outermost marker centres on both model diagonals were measured. This distance was determined as the mean of the distances between the points on the outer/inner edges of the outermost markers.

**Figure 12:**
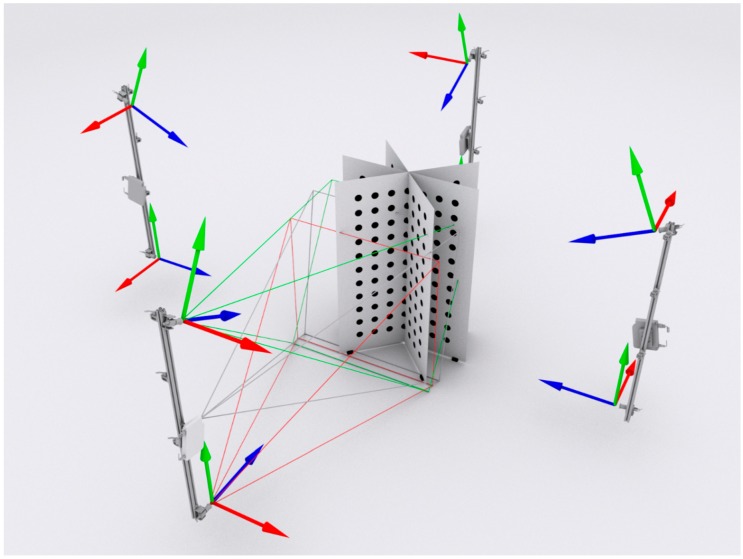
Calibration artefact positions employed in the validation analysis.

**Figure 13:**
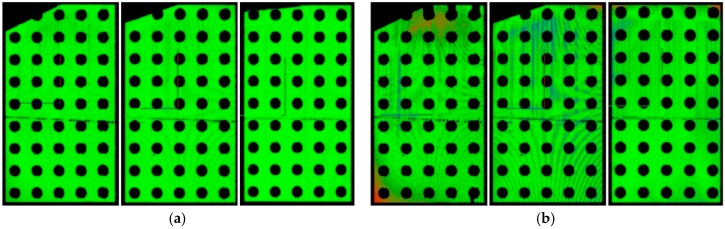

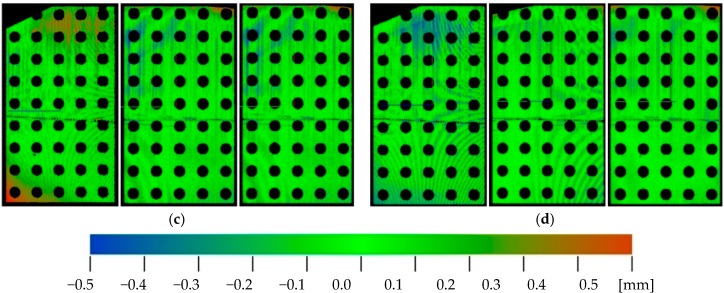
Examples of RMS errors obtained by plane fitting for a single DMH, for stable and rotating calibration artefact: (**a**) 0.0 rpm; (**b**) 4.0 rpm; (**c**) 9.0 rpm; (**d**) 14.0 rpm. In each panel, the outermost left and right images correspond to when the calibration artefact was oriented diagonally in the measurement volume. The middle image was taken when the calibration artefact was oriented perpendicular to the DMH. A portion of the upper left corner of the calibration artefact is missing from some of the images because part of the artefact was outside the calibrated volume.

For the validation, we used data originating from two frames in which the calibration artefact was oriented parallel to the diagonals of the measurement volume and one frame in which the calibration artefact was perpendicular to the DMH, as depicted in Figure 12. Such placement provides reliable estimates because, in most cases, extreme positions produce the highest measurement errors. Examples of the root mean square (RMS) errors obtained from plane fitting are presented in Figure 13. Table 3 lists the errors calculated for the analysed dynamic scenarios.

The calculated RMS errors are lower in the static case (0.0 rpm) than in the dynamic cases. In the dynamic cases, no correlation between the artefact rotation speed and RMS error is evident due to the relatively short shutter speed employed for camera image acquisition. However, we suggest that the influence of the rotation speed on the RMS error would be observable at higher rotation speeds.

### 5.2. Validation Using Human Subjects

To perform quantitative validation using human subjects, we employed a 3D body scanning system (OGX|MMS) and the body measurement algorithms developed by Markiewicz et al. [50] for reference. The OGX|MMS system was developed for whole-body static measurements as well as body dimension calculations. We performed parallel measurements of four subjects using our 4DBODY scanner and the reference OGX|MMS scanner [50]. Next, we calculated three body dimensions (namely, the waist, hip and chest girths) by applying OGX|MMS-validated algorithms to both measurements for each individual and compared the results. The average difference between the girths was 0.74 mm, while the maximum difference was 3.21 mm. After careful analysis, we ascertained that these differences originated from two main sources: the different postures adopted during measurement by the 4DBODY and OGX|MMS systems and the measurement uncertainties of both systems. When we performed the same comparison on a mannequin, the average and maximum differences were 0.27 mm and 0.38 mm, respectively, leading us to conclude that the measurement uncertainty is less than 0.5 mm. However, it is very difficult to obtain this level of uncertainty when using actual human subjects.

**Figure 14:**
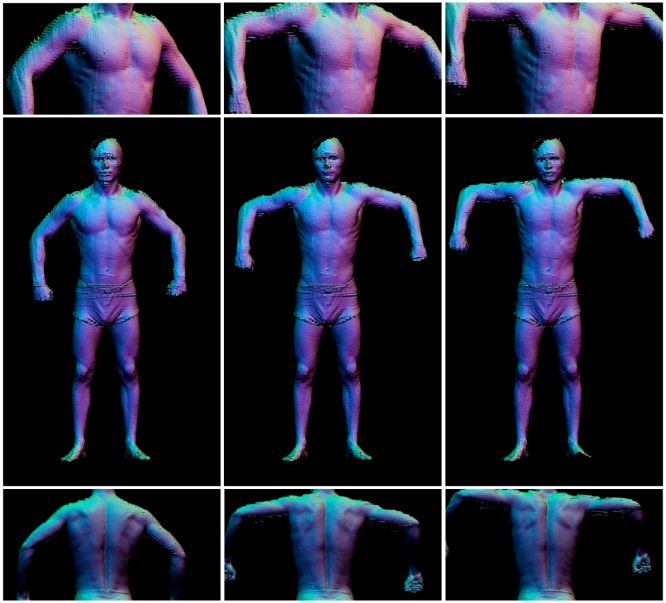
Three frames depicting a subject raising his shoulders. **Middle row**: full images; **top and bottom rows**: zoomed-in portions of the images in the **middle row**.

**Figure 15:**
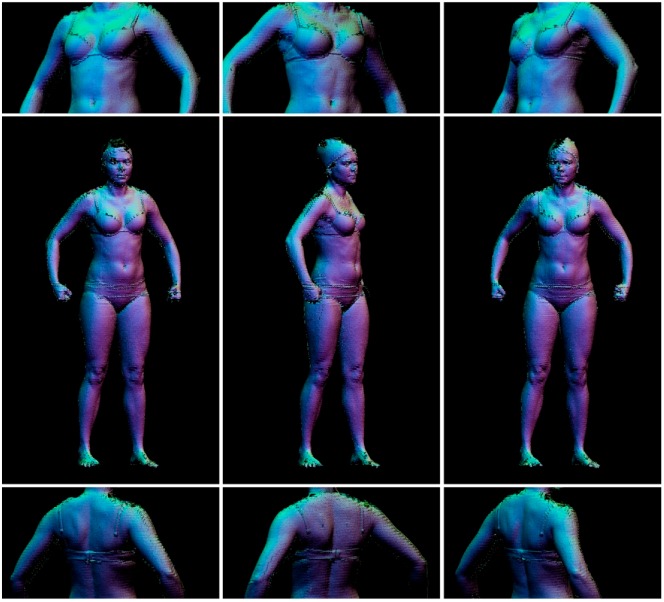
Three frames depicting a subject while turning her hips. **Middle row**: full images; **top and bottom rows**: zoomed-in portions of the images in the **middle row**.

The 4DBODY system was then tested on several individuals while they performed various basic movements. Measurements were taken at a frequency of 120 Hz, although the geometry reconstruction process was approximately 100 times longer than the acquisition process. The measurement data for each frame consisted of eight directional clouds integrated into one coordinate system. Each single cloud consisted of about 500,000 points. In addition to its (*x*, *y*, *z*) coordinates, each point contained information about the intensity and normal vector. With a measurement frequency of 30–120 Hz, we collected approximately 4–16 GB of data per second. Each constituent cloud had an average point-to-point distance of 1.5 mm, whereas the average point-to-point distance of the multidirectional cloud was approximately 1.0 mm. These temporal and spatial cloud densities enabled highly effective observation and representation of anatomical structures. Still images demonstrating the exemplary results of the proposed system are presented in Figure 14 and Figure 15. Example animations are also attached to this paper.

## 6. Conclusions and Future Work

This paper presented a 4DBODY system developed at the Virtual Reality Techniques Division of the Faculty of Mechatronics, Warsaw University of Technology, Poland. This system can realize full human body measurements at frequencies up to 120 Hz and the output point clouds can provide up to four million points per frame. The spatial resolution is approximately 1.0 mm and the uncertainty is less than 0.5 mm in both static and dynamic cases. Each point contains information about its intensity (i) and normal vector (*nx*, *ny*, *nz*), in addition to the standard (*x*, *y*, *z*) coordinates.

These features make the 4DBODY system potentially usable for supporting medical diagnostics and medical rehabilitation monitoring process. The primary advantage of the proposed system is that it does not require physical markers to be attached to the measured human body. Thus, the proposed system is entirely non-invasive, which is crucial for numerous applications. Marker-less measurement also provides information about the geometry of the entire body, which cannot be deduced based on measurements performed only at certain points. The current system performance supports medical diagnosis and rehabilitation monitoring from the point of view of input data for analysis. However, to enable the 4DBODY system to reach its full potential, new analytical algorithms need to be developed. These algorithms should use the full information provided by the extensive surface details during motion.

The 4DBODY system could also be applied in computer graphics and entertainment because the output, which is a cloud of points, can easily be converted into a mesh data type. Furthermore, our system provides a low-cost, or at most medium-cost, solution for dynamic full body measurements. Although the system performance enables full HD rendering of measurement data, there is a lack of colour mapped on the measured geometry.

In future work, we plan to focus on improving the system accuracy and reducing the processing time. We are considering increasing the number of processing units and re-implementing the graphics processing unit-based algorithms to achieve pseudo real-time reconstruction. We are also contemplating using RGB cameras to add colour information to each measurement point. Furthermore, new methods for 4D data analysis in various applications should be developed and validated.

## Figures and Tables

**Table 1:** Comparison of 4D full-field acquisition techniques.

Technique	Advantages	Disadvantages
**Structured Light**	High capture frequency High resolution	Prone to noise
**Structure from Motion**	High capture frequency Not affected by ambient light	Time-consuming processing Low resolution in some areas
**Time of Flight**	Not affected by ambient light	Low capture frequency
**Laser Triangulation**	Not affected by ambient light	Low resolution

**Table 2:** Comparison of the 4DBODY system with the previously proposed systems.

	Amount of Data	Acquisition Frequency	Equipment Cost	Marker-Less System
**4DBODY**	+	+	+	+
**3dMD system**	+	+/-	-	+
**DIERS International GmbH system**	-	+/-	+/-	+
**System proposed by Collet et al.**	+	+/-	-	+
**Microsoft Kinect 2.0**	-	-	+	+
**VICON**	-	+	+/-	-

**Table 3:** Root mean square (RMS) errors for the analysed dynamic scenarios.

	Rotation Speed [rpm]			
	0.0	4.0	9.0	14.0
**Average RMS error of plane fitting [mm]**	0.17	0.22	0.25	0.23
**Average RMS error of the distance between the outermost marker centres [mm]**	0.21	0.27	0.23	0.23
